# Causal association between tea intake and risk for gout: a Mendelian randomization study

**DOI:** 10.3389/fgene.2023.1220931

**Published:** 2023-07-13

**Authors:** Xiao Liang, Jingjing Cai, Yuchao Fan

**Affiliations:** ^1^ Department of Anesthesiology, West China Hospital, Sichuan University, Chengdu, Sichuan, China; ^2^ Department of Anesthesiology, Sichuan Clinical Research Center for Cancer, Sichuan Cancer Hospital and Institute, Sichuan Cancer Center, Affiliated Cancer Hospital of University of Electronic Science and Technology of China, Chengdu, China

**Keywords:** gout, tea, mendelian randomization, genome-wide association study, causal association

## Abstract

**Background:** Gout, an increasingly prevalent form of inflammatory arthritis, is caused by the accumulation of uric acid crystals in joints, resulting in severe pain, swelling and stiffness that adversely affect physical, mental and emotional wellbeing. The management of gout requires a combination of medication and lifestyle modifications. Recent studies suggest that tea intake may reduce the risk of developing gout; however, further research is needed to establish a causal relationship.

**Methods:** In this study, we employed a bidirectional two-sample Mendelian randomization (MR) approach, utilizing genome-wide association study (GWAS) summary statistics, to investigate the causal association between increased tea intake and gout. We meticulously selected instrumental variables (IVs) based on rigorous criteria and employed five different MR methods. Heterogeneity was assessed using Cochran’s Q statistic, and pleiotropy was evaluated using the MR Egger intercept and MR-PRESSO tests. Weak IVs were identified using F values. The Phenoscanner database was consulted to exclude single nucleotide polymorphisms associated with confounding factors or outcomes.

**Results:** The study included one dataset related to tea intake (ukb-b-6066) and three datasets related to gout (ukb-b-12765, finn-b-M13_GOUT, and finn-b-GOUT_STRICT). Our forward MR analysis suggest a causal relationship between increased tea intake and reduced risk of gout in all three gout-related datasets [OR (95% CI): 0.9966 (0.9938–0.9993), *p* = 0.0167; 0.4842 (0.2683–0.8737), *p*-value = 0.0160; and 0.4554 (0.2155–0.9623), *p* = 0.0393, respectively]. The reveres MR showed increased risk of gout (ukb-b-12765) was significantly associated with low tea intake according to the IVW analysis [OR (95% CI): 0.0062 (0.0002–0.154), *p* = 0.0020]. However, this association was not observed in the Finn-b-M13_GOUT and Finn-b-GOUT_STRICT [OR (95% CI): 0.9992 (0.9909–1.0075), *p* = 0.8453 and OR (95% CI): 0.9996 (0.9932–1.0059), *p* = 0.8896, respectively]. No significant heterogeneity or potential pleiotropy was detected, and the possibility of weak IVs was also excluded.

**Conclusion:** Our MR analysis suggest a causal relationship between genetically predicted tea intake and a decreased risk of gout. These findings underscore the potential advantages of increasing tea intake for preventing gout. However, further research is needed to validate these results and elucidate the underlying mechanisms.

## Introduction

Gout is a common inflammatory arthritis resulting from an imbalance between uric acid production and excretion, leading to uric acid crystal buildup in the joints (Dalbeth et al., 2021). In 2015 and 2016, the gout affected around 3.9% United States adults (2.7% in women and 5.2% in men) (Chen-Xu et al., 2019), with a higher prevalence among men, the elderly (Singh and Gaffo, 2020), and those with obesity, hypertension, and diabetes, as well as those with high consumption of alcohol, red meat, and sugary drinks (Dalbeth et al., 2021). The prevalence of gout has exhibited a persistent upward trend throughout the 20th century, in concurrence with alterations in the age composition of the populace and a concomitant surge in the incidence of the metabolic syndrome and its related pathologies (Kuo et al., 2015; Chen-Xu et al., 2019).

The condition can cause severe pain, swelling, and stiffness in the affected joints, with sharp and intense pain that can hinder even slight joint movement or touch (Taylor et al., 2015). While the first metatarsophalangeal joint is the most commonly affected joint, gout can also impact other joints such as the ankle, knee, wrist, and fingers (Bursill et al., 2019). During an attack, the joint becomes hot, red, and swollen, impeding simple activities, and is accompanied by fever and chills, leading to discomfort. Gout attacks, lasting days or weeks, can recur frequently if left untreated. Moreover, its chronic nature and unpredictable attacks can have a significant impact on mental and emotional health, leading to anxiety, depression, social isolation, and reduced quality of life (Zhu et al., 2012; Rai et al., 2017a). Gout can also interfere with daily activities such as work, exercise, and hobbies, affecting overall wellbeing (Zhu et al., 2012). Additionally, gout increases the risk of other severe health problems such as kidney stones, chronic kidney disease, and cardiovascular disease (Bevis et al., 2018), further exacerbating the suffering and increasing the burden on healthcare and society (Kuo et al., 2015).

Gout can cause severe pain and negatively impact an individual’s physical, mental, and emotional wellbeing. To prevent and manage gout, a combination of lifestyle modifications and medication is necessary (Juraschek et al., 2016; Nielsen et al., 2017), including reducing the intake of purine-rich foods, sugary drinks, weight loss, and exercise, all of which can help reduce uric acid levels in the body (Richette et al., 2017; FitzGerald et al., 2020). Recent evidence suggests that tea may also be beneficial in reducing the risk of gout (Guo et al., 2023).

Tea is a rich source of flavonoids, which possess anti-inflammatory and antioxidant properties (Trevisanato and Kim, 2000), and may help reduce uric acid levels and inflammation in the body (Chen et al., 2022; Liao et al., 2022). Although the precise mechanism underlying the potential association between tea intake and gout is not fully understood, several hypotheses have been proposed. One theory is that tea flavonoids may inhibit xanthine oxidase, an enzyme involved in uric acid production (Jatuworapruk et al., 2014), while another is that tea flavonoids may improve uric acid excretion by the kidneys (Chen et al., 2015; Feng et al., 2022). However, since observational studies may have numerous confounding factors, large randomized controlled trials are required to determine the effects of tea intake on gout (Zhang et al., 2017).

Mendelian randomization (MR) is a method that utilizes genetic variations as instrumental variables to investigate the causal relationship between a modifiable risk factor and an outcome of interest (Davey Smith and Hemani, 2014). One of the key advantages of MR is its ability to overcome confounding and reverse causation, which are common issues in observational studies (Skrivankova et al., 2021). MR studies also offer several other benefits, including the ability to assess the effects of lifelong exposure to a risk factor and the ability to examine the effects of modifiable risk factors on multiple outcomes (Sanderson, 2021). To explore the relationship between increased tea intake and gout risk, we conducted a two-sample MR study.

## Methods

### Study design

**FIGURE 1:**
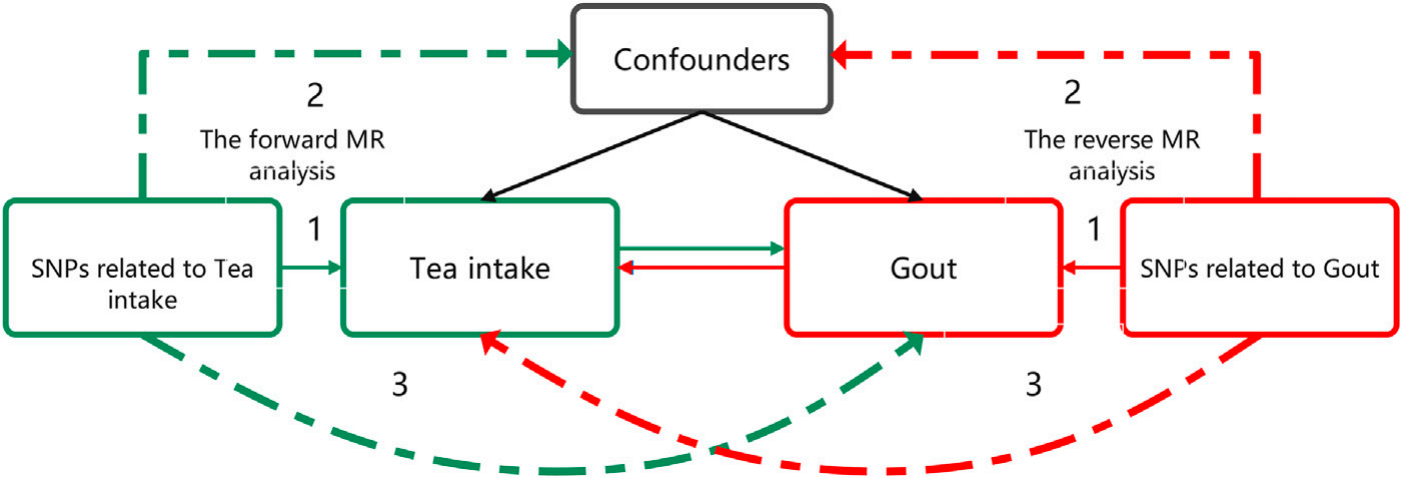
Bidirectional MR study design examining the causal effect of tea intake on gout. The green analysis investigates the causal effect of tea intake levels increasing as the exposure on gout risk as the outcome, while the red analysis examines the reverse association. The genetic variant should meet three criteria: 1) The genetic variant is associated with the exposure. 2) The genetic variant is not associated with any confounders of the exposure-outcome association. 3) The genetic variant does not affect outcome, except possibly through association with exposure. MR, Mendelian randomization.

This investigation employed a bidirectional two-sample MR approach utilizing genome-wide association study (GWAS) summary statistics to examine the association between increased tea intake and gout (Figure 1). In the forward MR analysis, tea intake was considered as the exposure and risk for gout as the outcome. Conversely, in reverse MR analysis, risk for gout was regarded as the exposure and tea intake as the outcome.

The objective of the study was to evaluate the causal association between tea intake and gout incidence as exposure and outcome, respectively, without necessitating ethical approval as publicly available data was utilized. The genetic variation was utilized to assess the causal effect of exposure on outcome. The fundamental requirements for genetic variation to fulfill the instrumental variable assumptions in this research are as follows: 1) The genetic variant must be associated with the exposure; 2) the genetic variant must not be associated with any confounders of the exposure-outcome association; and 3) the genetic variant should not influence the outcome, except possibly via its association with the exposure.

### Data source

This investigation utilized genetic associations from independent GWAS datasets of the same ancestral population to circumvent confounding factors. The estimation method for assessing the degree of population overlap between the exposure and outcome datasets is based on the consideration of the maximum potential proportion of overlap. Specifically, this method assumes that all cases in the outcome dataset are derived from the exposure dataset (i.e., maximal overlap). An overlap rate below 1% is considered to have minimal impact on the outcome and can be negligibly ignored (Pierce and Burgess, 2013).

## The forward MR

### Exposure data source

In the forward MR tea intake was utilized as an exposure. The GWAS dataset for tea intake (ukb-b-6066) was obtained from United Kingdom Biobank which encompassed 9,851,867 SNPs and a sample size of 447,485 individuals of European descent. The category of it is continuous and the value type is integer, cups/day. The Mean and Std. dev of the dataset are 3.49631 and 2.84255. The data pertaining to tea intake were sourced from two specific questionnaires: “Screenshot from touchscreen questionnaire used to capture field 1488, Res ID 100318”and “Touchscreen questionnaire ordering, validation and dependencies, Res ID 113241”. The questionnaires consisted of the following inquiry: “How many cups of tea do you drink each day? (Include black and green tea)” with a range of 0–99 cups. Participants were instructed to provide an average considering their intake over the past year.

### Outcome data source

In the forward MR risk for gout was utilized as the outcome. To enhance the reliability of our findings, we incorporated three datasets related to risk for gout in this study. In our study, cases of Risk for gout identified from the United Kingdom Biobank (ukb-b-12765) and Finngen (finn-b-M13_GOUT and finn-b-GOUT_STRICT) datasets were classified using the International Classification of Disease code 10 (ICD-10) M10, which is defined as a condition characterized by painful joint swelling due to urate crystal deposition. The specific codes assigned to ukb-b-12765, finn-b-M13_GOUT, and finn-b-GOUT_STRICT were M10.99, M10, and M10.0, respectively. M10.99 represents gout without a specified site, indicating the presence of gout without providing specific information about the affected site. This code is used when the site of gout occurrence is not explicitly specified. M10 is the code category encompassing various codes related to gout and its associated disorders. Codes within the M10 category are used to describe gout manifestations in different sites. M10.0 specifically refers to primary gout, which signifies gout where the underlying cause is not specified or clearly associated with any evident underlying disease. It is termed “primary” as it lacks specific triggers or causes but is attributed to abnormal uric acid metabolism.

All three datasets consist of individuals of European ancestry. The ukb-b-12765 dataset comprised 1,042 cases and a control group of 461,968 individuals, with a total of 9,851,867 SNPs. The finn-b M13_GOUT dataset consisted of 3,576 cases and a control group of 147,221 individuals, with a total of 16,380,152 SNPs. Similarly, the finn-b-GOUT_STRICT dataset included 1,699 cases and a control group of 216,239 individuals, with a total of 16,380,466 SNPs. A detailed summary of the datasets used in this study can be found in Table 1. Due to potential genetic differences between European populations and the Finnish population (Liu and Fu, 2015), the allele frequencies of significant SNPs in the three datasets were compared using Pearson chi-square test to account for the potential impact of genetic background differences on the results. The *p*-value <0.05 indicates a significant disparity in allele frequencies of the significant SNPs between the two groups. The R software was utilized for performing Pearson chi-square test.

**TABLE 1:** The detailed summary of GWAS datasets in MR analysis.

Trait	GWAS ID	Year	Cases	Controls	Simple size	SNPs	Population	Sex	Category
Tea intake	ukb-b-6066	2018	-	-	447,485	9,851,867	European	Males and Females	Continuous
Diagnoses - secondary ICD10: M10.99 Gout, unspecified (Site unspecified)	ukb-b-12765	2018	1,042	461,968	463,010	9,851,867	European	Males and Females	Binary
Gout	finn-b-M13_GOUT	2021	3,576	147,221	150,797	16,380,152	European	Males and Females	Binary
Gout, strict definition	finn-b-GOUT_STRICT	2021	1,699	216,239	217.938	16,380,466	European	Males and Females	Binary

GWAS, genome-wide association study; MR, mendelian randomization.

## The reverse MR

In reverse MR analysis, the exposed data were derived from the gout dataset above, while the outcome data were derived from the tea intake dataset above.

### Selection of instrumental variables

In this study, instrumental variables (IVs) were carefully selected based on rigorous criteria. Specifically, SNPs were considered as valid IVs if they showed a significant genome-wide association with the exposure (at P < 5e-8). Additionally, SNPs with a minor allele frequency (MAF) in the outcome greater than 0.01 and a linkage disequilibrium (LD) r2 of less than 0.001 within a 10,000 kb distance were chosen as IVs. We excluded SNPs that were associated with confounders or outcomes according to the Phenoscanner database (http://www.phenoscanner.medschl.cam.ac.uk/).

The proportion of variance explained by each SNP was calculated, and the F-statistic was computed to evaluate the strength of the IVs (Burgess et al., 2011). A value less than 10 indicated that the selected genetic variants were weak instrumental variables, which may lead to biased results. Therefore, we exercised caution when interpreting the results. The F-statistic was calculated using the formula $F=\frac{\left(N\;–\;k\;–\;1\right)}{k}\times \frac{R^{2}}{\left(1\;–\;R^{2}\right)}$ , where k represents the number of IVs employed in the analysis. The coefficient of determination (R2) was utilized as a metric to quantify the proportion of variance accounted for by individual SNPs. The *R* ^2^ was calculated using the formula $R^{2}=\frac{2{\times \beta }^{2}\times EAF\times \left(1-EAF\right)}{2{\times \beta }^{2}\times EAF\times \left(1-EAF\right)+2\times {SE}^{2}\times N\times EAF\times \left(1-EAF\right)}$ (Teslovich et al., 2010; Papadimitriou et al., 2020), where β represents the effect size of the SNP, EAF signifies the minor allele frequency, SE2 denotes the standard error of the effect size, and N denotes the total number of individuals in the GWAS study.

### Sensitivity analyses

To evaluate heterogeneity, we used the mr_egger and IVW methods to calculate Cochran’s Q statistic (Bowden et al., 2015; Burgess and Thompson, 2017). A *p*-value greater than 0.05 indicated no heterogeneity. “leave-one-out” analysis was conducted to assess the influence of individual SNPs on the causal effect of exposure on the outcome. In situations where heterogeneity existed, we employed a random-effects IVW method to estimate the causal association. Otherwise, we used a fixed-effects model. We tested the pleiotropy using the intercept *p*-value obtained from the MR Egger regression and global test *p*-value of MR-PRESSO, with *p* > 0.05 indicating no potential pleiotropy of IVs (Verbanck et al., 2018).

### Two-sample MR analysis

We conducted five different MR methods to investigate the causal association between tea intake and risk for gout. The Inverse Variance Weighting (IVW) method was our primary MR analysis, while MR Egger, weighted median, simple mode, and weighted mode methods were used as supplementary analyses. We considered a causal effect of exposure on the outcome to be significant if the *p*-value was less than 0.05. Effect estimates were reported as odds ratios (ORs) with corresponding 95% confidence intervals (CIs). We considered associations statistically significant if *p* values in IVW and MR-PRESSO methods were smaller than 0.05, and the results of MR-Egger, weighted median, Simple mode, and Weighted mode methods were consistent with IVW. R software (version 4.2.2) and R Package “TwoSampleMR” and “MRPRESSO” were used to conduct all statistical analyses in the MR analysis.

## Results

In the forward MR analysis, the Pearson chi-square test revealed significant differences in allele frequencies of the IVs: rs10741694, rs10752269, rs10764990, rs2783129, rs4410790, rs4817505, and rs713598 between the ukb-b-12765 and finn-b-M13_GOUT datasets. Therefore, these IVs were deemed unsuitable for further analysis (Tea intake as exposure on Gout finn-b-M13_GOUT as outcome) and were excluded. No significant differences in allele frequencies of the IVs were observed between the ukb-b-12765 and finn-b-GOUT_STRICT datasets (Supplementary Table S1). In the reverse MR analysis, no Pearson chi-square test was conducted as there was no one-to-one SNP correspondence between the three IVs in the ukb-b-12765 dataset and the other two datasets.

As there is no data overlap between the finn-b-M13_GOUT and finn-b-GOUT_STRICT datasets from the Finnish database and the UKB database, any observed differences in results cannot be attributed to this factor. Therefore, we evaluated the extent of sample overlap between Gout (ukb-b-12765) and Tea intake (ukb-b-6066). The findings revealed an overlap rate of 0.23% based on the maximum potential proportion of overlap, which falls below 1% and can be considered negligible in terms of its impact on the outcomes.

## The forward MR

### MR results of increased tea intake on risk for gout, unspecified (site unspecified) (ukb-b-12765)

We identified a total of 41 SNPs through the MR analysis process, out of which 22 SNPs were excluded for not meeting the inclusion criteria. One SNP, namely, rs2783129, was removed due to its palindromic nature with intermediate allele frequencies. The remaining 18 SNPs were deemed eligible for the analysis, as they did not demonstrate any association with gout or related confounders in Phenoscanner (Supplementary Table S2). and exhibited F-values greater than 10, indicating the absence of weak instruments (Supplementary Table S3).

**FIGURE 2:**
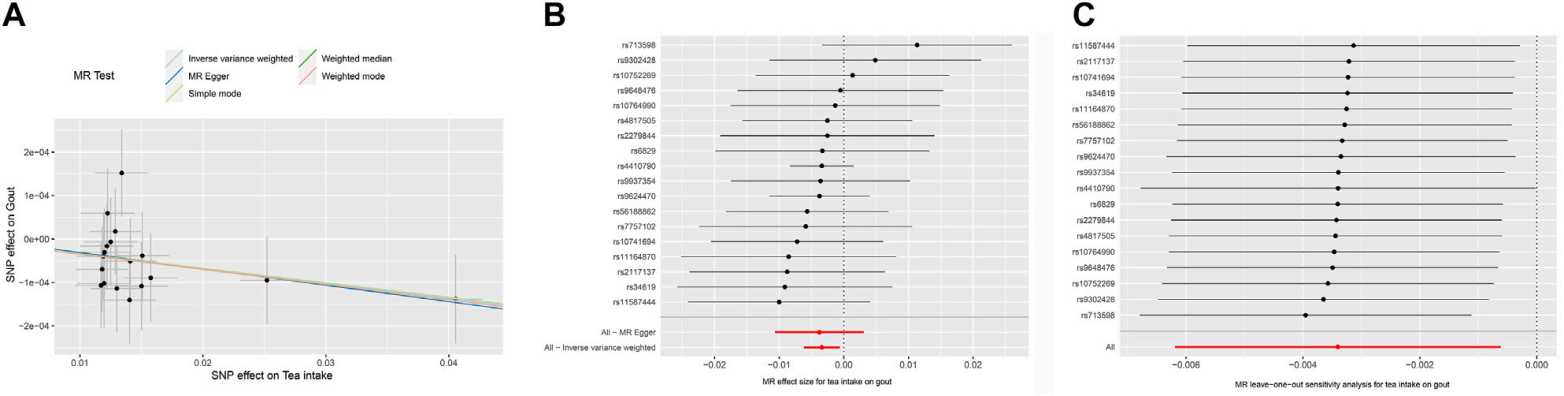
The MR analyse using Tea intake as exposure and gout (ukb-b-12765) risk as outcome. **(A)** Scatter plot in the MR analysis of Tea intake increasing on gout risk. The plot shows individual MR estimates, indicating that as the effect of individual SNPs on Tea intake increasing, so does the promotion of gout occurrence by individual SNPs. The *x*-axis indicates the SNP effect and standard error on Tea intake for each of the SNPs, while the *y*-axis shows the SNP effect and standard error on gout. The plot includes the regression line for mr_egger, weighted median, IVW, simple mode, and weighted mode. **(B)** Display of the forest plot for the single SNP analysis of Tea intake increasing on gout risk. The *x*-axis shows the MR effect size for Tea intake increasing on gout, while the *y*-axis illustrates the analysis for each of the SNPs. The dot and bar indicate the causal estimate and 95% CI of the association between Tea intake increasing and gout risk. **(C)** Presentation of the leave-one-out sensitivity analysis for the effect of Tea intake increasing SNPs on gout risk in the context of MR. The dot and bar indicate the estimate and 95% CI when a specific SNP is removed. IV, instrumental variant; IVW, inverse variance weighted; MR, Mendelian randomization; SE, standard error; SNP, single-nucleotide polymorphism.

The MR results demonstrated that genetically predicted increased tea intake were associated with a decreased risk of gout, as evidenced by the results obtained from the IVW models (OR and 95% CI: 0.9966, 0.9938–0.9993; *p* = 0.0167) (Table 2, Figures 2A, B). However, other models failed to demonstrate a significant association, but the direction of their results is consistent with the IVW method. Figure 2A displays the scatter plot illustrating the correlation between genetically predicted increased tea intake (ukb-b-12765) and gout risk, with each fitted line slope representing the combined effect obtained through various MR analysis methods. The forest plot depicting the magnitude of the MR effect for increased tea intake (ukb-b-12765) on gout risk is illustrated in Figure 2B. MR Egger and IVW in Cochran’s Q test did not reveal any significant heterogeneity among the 18 IVs in the gout GWAS, as evidenced by the absence of significant *p*-values (IVW *p* = 0.9633, MR Egger *p* = 0.9448) (Table 3). Furthermore, MR-Egger regression analyses and MR PRESSO results indicated no potential pleiotropy, further supporting the absence of confounding factors in these IVs (MR Egger *p* = 0.9084, MR-PRESSO *p* = 0.94) (Table 3). The leave-one-out analysis demonstrated that the causal association estimate was not influenced by the exclusion of any individual SNP (Figure 2C).

**TABLE 2:** MR analysis of exposure (Tea intake) with outcomes (Risk of Gout).

Exposure	Outcome	Method	Nsnp	b	SE	*p*-value	OR	Lower 95% CI.	Upper 95% CI
Tea intake (ukb-b-6066)	Gout, unspecified (ukb-b-12765)	MR Egger	18	−0.0038	0.0035	0.2975	0.9962	0.9894	1.0031
		Weighted median	18	−0.0035	0.0020	0.0760	0.9965	0.9927	1.0004
		Inverse variance weighted	18	−0.0034	0.0014	0.0167	0.9966	0.9938	0.9994
		Simple mode	18	−0.0034	0.0031	0.2904	0.9967	0.9907	1.0027
		Weighted mode	18	−0.0035	0.0021	0.1197	0.9965	0.9924	1.0007
	Gout (finn-b-M13_GOUT)	MR Egger	32	−2.0002	0.6308	0.0035	0.1353	0.0393	0.4659
		Weighted median	32	−1.3339	0.4016	0.0009	0.2635	0.1199	0.5788
		Inverse variance weighted	32	−0.7253	0.3012	0.0160	0.4842	0.2683	0.8737
		Simple mode	32	−1.1513	0.7648	0.1423	0.3162	0.0706	1.4158
		Weighted mode	32	−1.3863	0.4722	0.0062	0.2500	0.0991	0.6308
	Gout, strict definition (finn-b-GOUT_STRICT)	MR Egger	37	−2.4174	0.7782	0.0037	0.0892	0.0194	0.4098
		Weighted median	37	−1.2744	0.5154	0.0134	0.2796	0.1018	0.7678
		Inverse variance weighted	37	−0.7867	0.3818	0.0393	0.4554	0.2155	0.9623
		Simple mode	37	−1.0202	1.0143	0.3212	0.3605	0.0494	2.6321
		Weighted mode	37	−1.4144	0.5015	0.0078	0.2431	0.0910	0.6495

MR, mendelian randomization; SE, standard error; OR, odds ratio; CI, confidence intervals.

**TABLE 3:** The Heterogeneity and Pleiotropy test of MR analysis.

		Heterogeneity test		Pleiotropy test	
Exprosure	Outcome	IVW p	MR-egger p	MR-egger *p*	PRESSO *p*
Tea intake	Gout, unspecified (ukb-b-12765)	0.9633	0.9448	0.9084	0.9400
	Gout (finn-b-M13_GOUT)	0.2728	0.4580	0.0512	0.2560
	Gout, strict definition (finn-b-GOUT_STRICT)	0.2041	0.3828	0.1670	0.2300

IVW, inverse variance weighted; MR, Mendelian randomization.

### MR results of increased tea intake on risk for gout (finn-b-M13_GOUT)

We identified a total of 41 SNPs through the MR analysis process, out of which 2 SNPs were excluded for not meeting the inclusion criteria. One SNP, namely, rs2783129, was removed due to its palindromic nature with intermediate allele frequencies. Due to the significant disparities in allele frequencies of rs10741694, rs10752269, rs10764990, rs2783129, rs4410790, rs4817505, and rs713598 compared to the European population, they are deemed unsuitable for further analysis and have been excluded. The remaining 32 SNPs were deemed eligible for the analysis, as they did not demonstrate any association with gout or related confounders in Phenoscanner and exhibited F-values greater than 10, indicating the absence of weak instruments (Supplementary Table S3).

**FIGURE 3:**
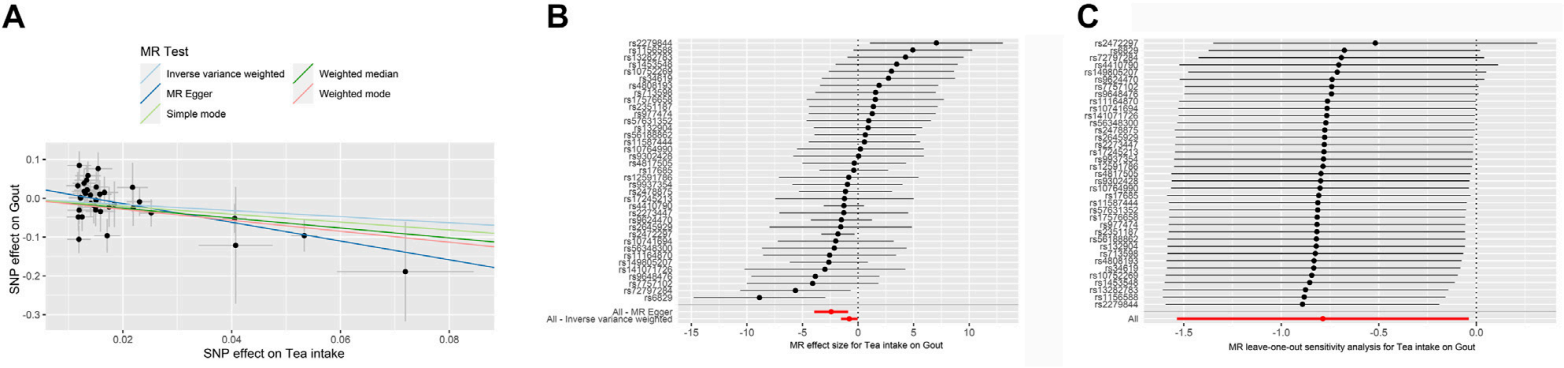
The MR analyse using Tea intake increasing as exposure and gout (finn-b-M13_GOUT) risk as outcome. **(A)** Scatter plot in the MR analysis of Tea intake increasing on gout risk. The plot shows individual MR estimates, indicating that as the effect of individual SNPs on Tea intake increasing, so does the promotion of gout occurrence by individual SNPs. The *x*-axis indicates the SNP effect and standard error on Tea intake for each of SNPs, while the *y*-axis shows the SNP effect and standard error on gout. The plot includes the regression line for mr_egger, weighted median, IVW, simple mode, and weighted mode. **(B)** Display of the forest plot for the single SNP analysis of Tea intake increasing on gout risk. The *x*-axis shows the MR effect size for Tea intake increasing on gout, while the *y*-axis illustrates the analysis for each of the SNPs. The dot and bar indicate the causal estimate and 95% CI of the association between Tea intake increasing and gout risk. **(C)** Presentation of the leave-one-out sensitivity analysis for the effect of Tea intake increasing SNPs on gout risk in the context of MR. The dot and bar indicate the estimate and 95% CI when a specific SNP is removed. IV, instrumental variant; IVW, inverse variance weighted; MR, Mendelian randomization; SE, standard error; SNP, single-nucleotide polymorphism.

The MR results demonstrated that genetically predicted increased tea intake were associated with a decreased risk of gout, as evidenced by the results obtained from the IVW models (OR and 95% CI: 0.4842, 0.2683–0.8737; *p* = 0.0160) (Table 2, Figures 3A, B). Additionally, there was a significant correlation observed with MR Egger, weighted median, and weighted mode (*p* = 0.0035, 0.0009, and 0.0062, respectively). Although Simple mode did not exhibit a significant correlation, its results align with the direction of the IVW method (Table 2). Figure 3A displays the scatter plot illustrating the correlation between genetically predicted increased tea intake (finn-b-M13_GOUT) and gout risk, with each fitted line slope representing the combined effect obtained through various MR analysis methods. The forest plot depicting the magnitude of the MR effect for increased tea intake (finn-b-M13_GOUT) on gout risk is illustrated in Figure 3B. MR Egger and IVW in Cochran’s Q test did not reveal any significant heterogeneity among the 32 IVs in the gout GWAS, as evidenced by the absence of significant *p*-values (IVW *p* = 0.2725, MR Egger *p* = 0.4580) (Table 3). Furthermore, MR-Egger regression analyses and MR PRESSO results indicated no potential pleiotropy, further supporting the absence of confounding factors in these IVs (MR Egger *p* = 0.0512, MR-PRESSO *p* = 0.2560) (Table 3). The leave-one-out analysis demonstrated that the causal association estimate was not influenced by the exclusion of any individual SNP (Figure 3C).

### MR results of increased tea intake on risk for gout, strict definition (finn-b-GOUT_STRICT)

We identified a total of 41 SNPs through the MR analysis process, out of which 3 SNPs were excluded for not meeting the inclusion criteria. One SNP, namely, rs2783129, was removed due to its palindromic nature with intermediate allele frequencies. The remaining 37 SNPs were deemed eligible for the analysis, as they did not demonstrate any association with gout or related confounders in Phenoscanner and exhibited F-values greater than 10, indicating the absence of weak instruments (Supplementary Table S3).

**FIGURE 4:**
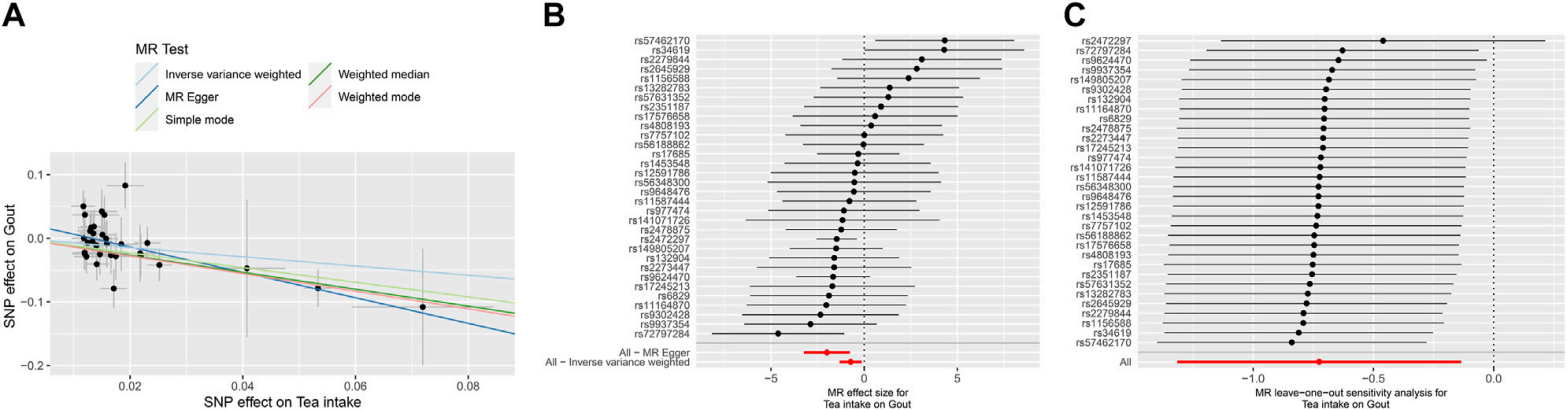
The MR analyse using Tea intake increasing as exposure and gout (finn-b-GOUT_STRICT) risk as outcome. **(A)** Scatter plot in the MR analysis of Tea intake increasing on gout risk. The plot shows individual MR estimates, indicating that as the effect of individual SNPs on Tea intake increasing, so does the promotion of gout occurrence by individual SNPs. The *x*-axis indicates the SNP effect and standard error on Tea intake for each of the SNPs, while the *y*-axis shows the SNP effect and standard error on gout. The plot includes the regression line for mr_egger, weighted median, IVW, simple mode, and weighted mode. **(B)** Display of the forest plot for the single SNP analysis of Tea intake increasing on gout risk. The *x*-axis shows the MR effect size for Tea intake increasing on gout, while the *y*-axis illustrates the analysis for each of the SNPs. The dot and bar indicate the causal estimate and 95% CI of the association between Tea intake increasing and gout risk. **(C)** Presentation of the leave-one-out sensitivity analysis for the effect of Tea intake increasing SNPs on gout risk in the context of MR. The dot and bar indicate the estimate and 95% CI when a specific SNP is removed. IV, instrumental variant; IVW, inverse variance weighted; MR, Mendelian randomization; SE, standard error; SNP, single-nucleotide polymorphism.

The MR results demonstrated that genetically predicted increased tea intake were associated with a decreased risk of gout, as evidenced by the results obtained from the IVW models (OR and 95% CI: 0.4554, 0.2155–0.9623; *p* = 0.0393) (Table 2, Figures 4A, B). Additionally, there was a significant correlation observed with MR Egger, weighted median, and weighted mode (*p* = 0.0037, 0.0393, and 0.0078, respectively). Although Simple mode did not exhibit a significant correlation, its results align with the direction of the IVW method (Table 2). Figure 4A displays the scatter plot illustrating the correlation between genetically predicted increased tea intake (finn-b-GOUT_STRICT) and gout risk, with each fitted line slope representing the combined effect obtained through various MR analysis methods. The forest plot depicting the magnitude of the MR effect for increased tea intake (finn-b-GOUT_STRICT) on gout risk is illustrated in Figure 4B. MR Egger and IVW in Cochran’s Q test did not reveal any significant heterogeneity among the 37 IVs in the gout GWAS, as evidenced by the absence of significant *p*-values (IVW *p* = 0.2041, MR Egger *p* = 0.3828) (Table 3). Furthermore, MR-Egger regression analyses and MR PRESSO results indicated no potential pleiotropy, further supporting the absence of confounding factors in these IVs (MR Egger *p* = 0.1670, MR-PRESSO *p* = 0.23) (Table 3). The leave-one-out analysis demonstrated that the causal association estimate was not influenced by the exclusion of any individual SNP (Figure 4C).

## The reverse MR

In the subsequent analysis, we proceeded with a reverse MR investigation to explore the potential causal association between the risk of gout and tea intake levels. We initially harmonized the data with the Tea intake dataset, obtaining three gout-associated genetic variants from the gout, unspecified (ukb-b-12765) dataset and six genetic variants from the Finn-b-M13_GOUT and Finn-b-GOUT_STRICT datasets, which served as IVs (Supplementary Table S3). To ensure the validity of our analysis, we employed MR-PRESSO to identify and address any significant pleiotropy among the independent IVs, resulting in no noteworthy pleiotropic effects. Moreover, the MR-Egger intercept *p*-value indicated the absence of significant pleiotropy. Furthermore, both the MR Egger and IVW methods demonstrated no substantial heterogeneity among the independent IVs based on Cochran’s Q statistics (Supplementary Table S4). Consequently, the IVW fixed method, serving as the primary statistical approach in our reverse MR study, was employed to evaluate the potential causal relationship between gout risk and tea intake.

The IVW analysis results provided evidence supporting a causal relationship between a increased risk of unspecified gout (ukb-b-12765) and low tea intake, with an OR of 0.0062 (95% CI: 0.0002–0.1547) and a *p*-value of 0.0020 (Supplementary Table S5; Supplementary Figures S1A, B). However, when examining the Finn-b-M13_GOUT and Finn-b-GOUT_STRICT databases, no significant causal relationship was observed for gout risk and tea intake, as indicated by ORs of 0.9992 (95% CI: 0.9909–1.0075; *p* = 0.8453) and 0.9996 (95% CI: 0.9932–1.0059; *p* = 0.8896), respectively (Supplementary Table S5; Supplementary Figures S2, S3A, B). Furthermore, in the leave-one-out sensitivity analysis, each SNP demonstrated heterogeneity when compared to the other SNPs, as shown in Supplementary Figure S1–S3C.

## Discussion

The present study aimed to investigate the causal association between increased tea intake and the risk of gout, utilizing a bidirectional two-sample MR approach. To the best of our knowledge, this study is the first to employ MR methodology to explore the link between tea intake and gout risk. Our results suggest that a correlation between genetically predicted increased tea intake levels and an decreased susceptibility to risk of gout. The MR analysis employed in this study revealed non-significant heterogeneity and pleiotropy for the IVs, while the utilization of PhenoScanner facilitated the identification of SNPs that might be associated with pleiotropy, further confirming the validity of the IVs employed in this study. Notably, all the IVs demonstrated adequate strength, with F values exceeding 10. Furthermore, the utilization of three gout-associated datasets from two distinct biobanks in this investigation, all of which produced consistent outcomes, undoubtedly provides robust validation for our findings. Specifically, the inclusion of patients with a strict diagnosis of GOUT in the finn-b-GOUT_STRICT dataset adds further credibility to our findings that an increased intake of tea is associated with a reduced risk of gout.

We conducted additional reverse MR analyses. The reverse MR analysis revealed that an elevated risk of Gout, unspecified (ukb-b-12765) may lead to lower tea intake. This suggests a potential reverse causal effect between gout risk and tea intake, indicating a bidirectional relationship between gout risk and tea intake. On the one hand, a lifestyle characterized by increased tea intake may potentially reduce the risk of gout. Conversely, individuals with a higher susceptibility to gout may inherently intake less tea, thereby further increasing their risk of developing this condition. However, we did not observe the reverse causal effect in the other two Finnish datasets. There could be several possible reasons for this. Firstly, it is plausible that the reverse causal effect between gout, unspecified (ukb-b-12765) and tea intake may represent a false positive result due to chance or methodological limitations, which prompted us to incorporate multiple datasets related to gout for analysis. Furthermore, the other two datasets are derived from Finnish populations in Europe, which may exhibit dissimilarities compared to the general European population (Liu and Fu, 2015). Moreover, our findings indicate significant differences in allele frequencies of certain validated SNPs between the UKB dataset and the Finn dataset. Given the potential bottleneck encountered by the ancestors of the FIN populace approximately 10,000–20,000 years ago, it is plausible that the occurrence of the founder effect could be observed in these particular SNPs exhibiting substantial allele frequencies disparities (Liu and Fu, 2015). Additionally, these findings imply the existence of possible distinct biological pathways or mechanisms that are specific to gout susceptibility between non-Finnish Europeans and individuals of European descent with Finnish heritage.

Given that the genetic variations associated with Tea intake (ukb-b-6066) and unspecified gout (ukb-b-12765) were obtained from participants in the United Kingdom Biobank, it is important to consider potential bias in the MR estimates due to the likelihood of overlapping samples in the studie (Burgess et al., 2016). Therefore, we employed the maximum proportion overlap method to calculate the sample overlap rate between the two databases. The analysis revealed a sample overlap rate of 0.23%, which is below 1% and thus has minimal impact on the results (Pierce and Burgess, 2013). In light of a substantial overlap between the exposure and outcome data in the United Kingdom Biobank study, the F statistic was computed as a metric to assess the instrument’s robustness and efficacy in the analyses (Burgess et al., 2016). The IVs’ F-values in our analysis all exceeded 10, providing further reduction in potential bias.

Previous studies investigating the association between tea intake and the risk of gout have reported inconsistent results. Some studies have reported an inverse association (Feng et al., 2022; Guo et al., 2023), while others have found no significant association or even a positive association (Choi and Curhan, 2007; Jatuworapruk et al., 2014). However, the reliability of these studies is questionable due to limitations such as confounding, measurement error, and reverse causality (Sekula et al., 2016). Our findings suggest that increased tea intake may reduce the risk of gout. Specifically, our study found a modest but statistically significant inverse association between genetically predicted increases in tea intake and the risk of gout.

While the biological mechanisms underlying this relationship are not fully understood, it is hypothesized that tea’s polyphenols, such as theaflavins, gallic acid, and other relevant compounds, which have antioxidant and anti-inflammatory properties (Chen et al., 2022; Liao et al., 2022), may play a protective role against gout by reducing uric acid production (Chen et al., 2015; Feng et al., 2022) or inhibiting inflammation (Jatuworapruk et al., 2014; Ohishi et al., 2016). Catechins possess antioxidative and anti-inflammatory properties, playing a positive role in regulating the disturbance of uric acid metabolism. In terms of uric acid metabolism regulation, catechins inhibit the activity of xanthine oxidase, reducing the excessive production of uric acid in the liver. They also modulate the expression of uric acid transporters, including URAT1, OAT1, OAT3, ABCG2, and GLUT9, to achieve a balance between uric acid secretion and reabsorption in the kidneys and intestines. Additionally, catechins effectively prevent inflammation caused by urate crystals. Dietary intake of catechins reduces the risk of hyperuricemia (Jatuworapruk et al., 2014; Wu et al., 2020). Moreover, catechins exhibit potent free radical scavenging activity, significantly reducing the secretion of IL-1β and IL-6 in C57BL/6 mice induced by MSU crystals, while inhibiting the activation of the NLRP3 inflammasome, thus effectively reducing the likelihood of developing gout in patients (Jhang et al., 2015). Theaflavins exert their anti-gout effects by downregulating the gene and protein expression of GLUT9 and URAT1 (Chen et al., 2023). On the other hand, gallic acid exhibits anti-inflammatory effects by inhibiting the activation of the NLRP3 inflammasome, suppressing subsequent caspase-1 activation, and reducing the secretion of IL-1β. Further investigations have demonstrated that gallic acid inhibits the generation of ROS, thereby restricting the activation and pyroptotic response of the NLRP3 inflammasome dependent on the Nrf2 signaling pathway. These findings suggest the potential of gallic acid as a therapeutic agent for the treatment of gouty arthritis (Lin et al., 2020).

Uric acid is a natural metabolic byproduct, that is, typically eliminated from the body via renal excretion. However, excessive uric acid production or reduced elimination can lead to its accumulation in the body, resulting in gout attacks. The excess uric acid forms crystals that deposit in joints and surrounding tissues, triggering an inflammatory response and causing symptoms of gout (Clebak et al., 2020). Tea and its compounds have shown inhibitory effects on xanthine oxidase and adenosine deaminase enzyme activity in animal models (Zhu et al., 2017; 2018). These enzymes play a crucial role in purine metabolism, and their inhibition can effectively reduce the accumulation of serum uric acid, thereby exerting an anti-hyperuricemic effect. Additionally, tea compounds have been shown to modulate the function of uric acid transporters in the kidneys, facilitating renal excretion of uric acid (Chen et al., 2015; Zhu et al., 2018). Moreover, tea and its compounds, particularly polyphenols with potent antioxidant properties, have demonstrated potential in ameliorating uric acid-induced inflammation of endothelial cells, joints, and other tissues. Numerous studies conducted on cells, animals, and humans have provided substantial evidence supporting the notion that tea possesses inhibitory effects on gout inflammation (Bahorun et al., 2010; Jhang et al., 2016; Chen and Xu, 2018; Lee et al., 2019).

There is currently some epidemiological evidence controversy regarding the relationship between tea intake and gout. In a health and nutrition survey conducted among adult participants in the United States, a total of 14,314 individuals without a history of gout or the use of allopurinol or uridine drugs were followed up for 6 years. The study findings showed that increased tea intake did not lead to a decrease in serum uric acid levels in both American males and females (Choi and Curhan, 2007) However, a prospective study involving 9,400 adults (≥40 years old) in Korea revealed a significant association between tea intake and serum uric acid levels in both males and females, although it was unrelated to the risk of hyperuricemia (Bae et al., 2015).

However, a randomized crossover study conducted in Japan indicated that green tea catechins may increase the excretion of uric acid, xanthine, and hypoxanthine (Kawakami et al., 2021). Another cross-sectional study conducted in China suggested a significant association between high levels of tea intake and a reduced risk of hyperuricemia in males, whereas no relationship was found between tea intake and serum uric acid levels in females (Li et al., 2015). Additionally, a prospective cohort study found that consuming at least 2–3 cups of tea per day was associated with a reduced risk of kidney stones, with uric acid stones accounting for 7%–10% of cases (Chen et al., 2019; Barghouthy et al., 2021). Recently, a prospective cohort study based on the United Kingdom Biobank demonstrated a strong nonlinear association between tea intake and the risk of gout, with a significant risk reduction observed by consuming 6 cups of tea per day (Guo et al., 2023). These findings support the notion that promoting tea intake could be a simple and cost-effective approach to reducing the burden of gout, particularly in high-risk individuals (Feng et al., 2022; Guo et al., 2023). However, it is crucial to consider the potential adverse effects of excessive tea intake, such as insomnia, anxiety, and cardiovascular effects (Hayat et al., 2015). Thus, the intake of tea should be balanced and within recommended levels. Future studies should aim to replicate our findings in diverse populations and investigate the specific mechanisms underlying the association between tea intake and gout risk. In addition, it is crucial to determine the optimal dose and duration of tea intake required to reduce the risk of gout and to investigate the potential adverse effects of excessive tea intake.

Despite providing insights into the potential causal relationship between tea intake and gout risk, our study has several limitations that need to be acknowledged. Firstly, like all MR studies, our findings are based on MR assumptions, which may have limitations. Although we conducted multiple sensitivity analyses to assess the robustness of our results, the possibility of unmeasured confounding factors influencing our findings cannot be ruled out (Emdin et al., 2017; Richmond and Davey Smith, 2022). Secondly, the tea intake dataset of our study relied on self-reported data from GWAS studies, which may suffer from recall bias and may not reflect actual tea intake accurately. Meanwhile, tea can be categorized into different types, such as green tea, black tea, and white tea, based on variations in processing methods and procedures. Each type of tea may have distinct or varying concentrations of chemical constituents, resulting in different effects on gout (Chen et al., 2022). Our study solely utilized a dataset related to tea intake, specifically incorporating black and green tea, without analyzing other specific types of tea. Further investigation into the effects of other specific types of tea would contribute to a more comprehensive understanding of the relationship between tea intake and gout. Thirdly, the generalizability of our findings to other populations may be limited as our study was based on data from individuals of European ancestry. Furthermore, it is worth noting that two of the three datasets analyzed in our study, which pertain to gout, were derived from Finnish populations. European populations can be classified into Finnish and non-Finnish Europeans (Liu and Fu, 2015). Furthermore, our study findings also demonstrate differences in allele frequencies of certain significant SNPs between the FINN and UKB datasets, indicating that potential genetic background disparities may still influence our results. Morever, although our findings indicate a reduced risk of gout with increased tea intake across the three associated datasets, caution must be exercised in interpreting these results due to variances in inclusion criteria and potential heterogeneity in the specific mechanisms and sites of gout manifestation within these datasets. Consequently, a prudent approach is necessary when considering the implications of our analysis. While tea intake may be linked to a diminished risk of gout, the precise underlying mechanisms and the influence of tea intake on risk of gout could exhibit individual variations. Therefore, further research is warranted to deepen our understanding of the complex relationship between tea intake and risk of gout, as well as its differential impact on gout occurrence and pathogenesis across diverse populations. Lastly, our study suggests a potential causal relationship between tea intake and gout risk; however, it is important to note that other lifestyle factors such as diet and physical activity may also contribute to the development of gout. Thus, further research is necessary to explore the role of tea intake in the context of other lifestyle factors that may impact gout risk (Rai et al., 2017b; Hui et al., 2017).

## Conclusion

In summary, our MR analysis suggest a causal association between genetically predicted increased tea intake and a reduced risk of gout. Our study contributes to the existing literature on the potential health benefits of tea intake and underscores the value of MR analysis in elucidating causal relationships between exposures and outcomes. Nonetheless, our study has limitations that warrant consideration when interpreting the findings. Further investigation is necessary to validate our results and to investigate the underlying biological mechanisms.

## Data Availability

The original contributions presented in the study are included in the article/Supplementary Material, further inquiries can be directed to the corresponding author.
