# Supplementary material for: Causal association between tea intake and risk for gout: a Mendelian randomization study
Source: Front Genet. 2023 Jul 13;14:1220931. doi: 10.3389/fgene.2023.1220931 (PMC10374259; doi:10.3389/fgene.2023.1220931)
Supplement: Supplementary file 1 [file DataSheet1.PDF]

Figure S1 The reverse MR analyse using gout (ukb-b-12765) risk as exposure and Tea intake as outcome. (A) Scatter plot in the MR analysis of increasing gout risk on Tea intake level. The plot shows individual MR estimates, indicating that as the effect of individual SNPs on increasing gout risk, so does the promotion of Tea intake level by individual SNPs. The x-axis indicates the SNP effect and standard error on gout risk for each of the SNPs, while the y-axis shows the SNP effect and standard error on Tea intake. The plot includes the regression line for mr_egger, weighted median, IVW, simple mode, and weighted mode. **(B)** Display of the forest plot for the single SNP analysis of gout risk on Tea intake level. The x-axis shows the MR effect size for increasing gout risk on Tea intake level, while the y-axis illustrates the analysis for each of the SNPs. The dot and bar indicate the causal estimate and 95% CI of the association between Tea intake increasing and gout risk. **(C)** Presentation of the leave-one-out sensitivity analysis for the effect of gout risk SNPs on Tea intake levels in the context of MR. The dot and bar indicate the estimate and 95% CI when a specific SNP is removed. IV, instrumental variant; IVW, inverse variance weighted; MR, Mendelian randomization; SE, standard error; SNP, single - nucleotide polymorphism;

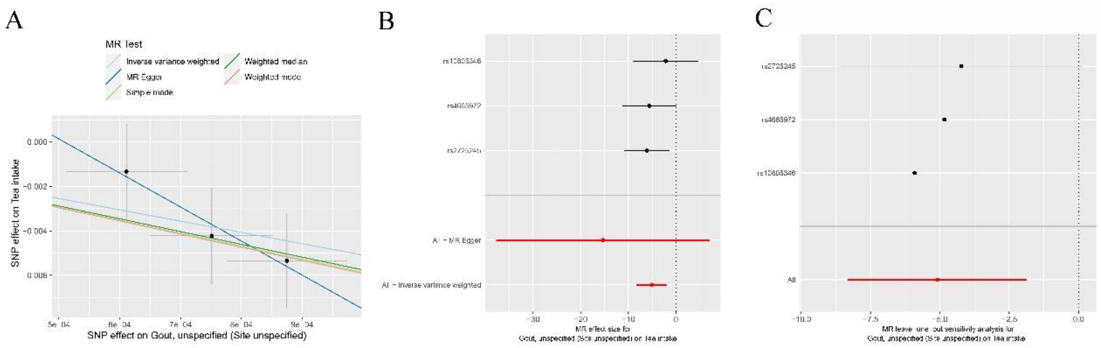

Figure S2 The reverse MR analyse using gout (finn-b-M13_GOUT) risk as exposure and Tea intake as outcome. (A) Scatter plot in the MR analysis of increasing gout risk on Tea intake level. The plot shows individual MR estimates, indicating that as the effect of individual SNPs on increasing gout risk, so does the

promotion of Tea intake level by individual SNPs. The x-axis indicates the SNP effect and standard error on gout risk for each of the SNPs, while the y-axis shows the SNP effect and standard error on Tea intake. The plot includes the regression line for mr_egger, weighted median, IVW, simple mode, and weighted mode. **(B)** Display of the forest plot for the single SNP analysis of gout risk on Tea intake level. The x-axis shows the MR effect size for increasing gout risk on Tea intake level, while the y-axis illustrates the analysis for each of the SNPs. The dot and bar indicate the causal estimate and 95% CI of the association between Tea intake increasing and gout risk. **(C)** Presentation of the leave-one-out sensitivity analysis for the effect of gout risk SNPs on Tea intake levels in the context of MR. The dot and bar indicate the estimate and 95% CI when a specific SNP is removed. IV, instrumental variant; IVW, inverse variance weighted; MR, Mendelian randomization; SE, standard error; SNP, single－nucleotide polymorphism;

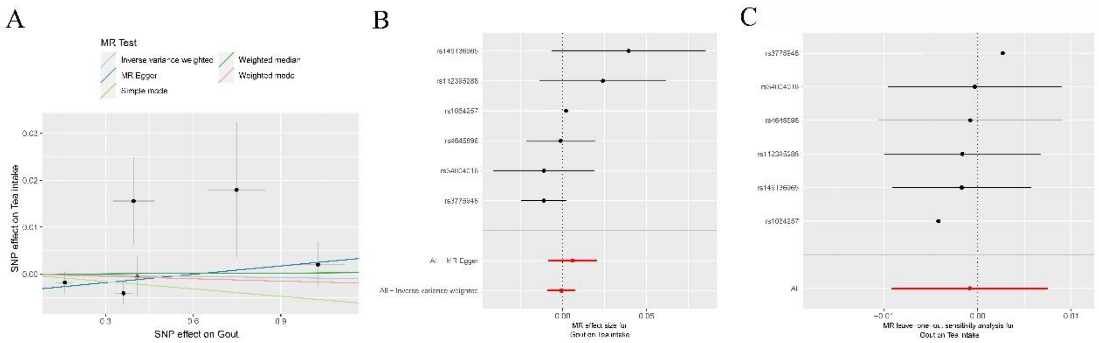

Figure S3 The reverse MR analyse using gout (finn-b-GOUT_STRICT) risk as exposure and Tea intake as outcome. (A) Scatter plot in the MR analysis of increasing gout risk on Tea intake level. The plot shows individual MR estimates, indicating that as the effect of individual SNPs on increasing gout risk, so does the

promotion of Tea intake level by individual SNPs. The x-axis indicates the SNP effect and standard error on gout risk for each of the SNPs, while the y-axis shows the SNP effect and standard error on Tea intake. The plot includes the regression line for mr_egger, weighted median, IVW, simple mode, and weighted mode. **(B)** Display of the forest plot for the single SNP analysis of gout risk on Tea intake level. The x-axis shows the MR effect size for increasing gout risk on Tea intake level, while the y-axis illustrates the analysis for each of the SNPs. The dot and bar indicate the causal estimate and 95% CI of the association between Tea intake increasing and gout risk. **(C)** Presentation of the leave-one-out sensitivity analysis for the effect of gout risk SNPs on Tea intake levels in the context of MR. The dot and bar indicate the estimate and 95% CI when a specific SNP is removed. IV, instrumental variant; IVW, inverse variance weighted; MR, Mendelian randomization; SE, standard error; SNP, single - nucleotide polymorphism;

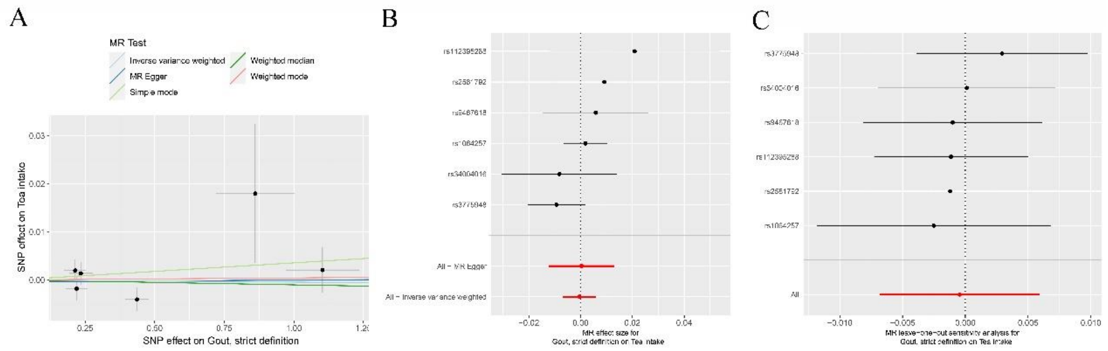

Table S1. Pearson chi-square test for allele frequencies of the IVs between UKB and Finn detasets.

SNP	P.value of chi-square test for ukb-b-12765 vs.finn-b-M13_GOUT	P.value of chi-square test for.ukb-b-12765 vs.finn-b-GOUT_STRICT
rs10741694	5.84E-11	1
rs10752269	4.45E-16	1
rs10764990	1.50E-05	1
rs11164870	0.175201472	1
rs11587444	0.573683208	1
rs2279844	0.412256285	1
rs2783129	0.039851191	1
rs34619	0.715453657	1
rs4410790	0.044555757	1
rs4817505	0.014885463	1
rs56188862	0.234926497	1
rs6829	0.863634262	1
rs713598	0.002257153	1
rs7757102	0.545491467	1
rs9302428	0.980761855	1
rs9624470	0.079694979	1
rs9648476	0.351011834	1
rs9937354	0.924476554	1

Table S2. Details of the data obtained by searching for instrumental variables in Phenoscanner

Variant	Pos	Effect allele	Other allele	Trait	Type	PMID	Beta	P	N	Unit	A1 freq (EUR):	Function	Gene
rs10741694	chr11:16286183	C	T	Vascular or heart problems diagnosed by doctor: high blood pressure	Diseases and traits	UKBB	-0.007586	1.18E-11	336683	risk diff	0.6233	intron	SOX6
				Vascular or heart problems diagnosed by doctor: none of the above	Diseases and traits	UKBB	0.007428	1.00E-10	336683	risk diff			
				Self-reported hypertension	Diseases and traits	UKBB	-0.006779	8.02E-10	337159	risk diff			
				Femoral neck mineral density	Diseases and traits	26367794	-0.03814	9.94E-07	32735	Z-score			
				Medication for cholesterol, blood pressure or diabetes: blood pressure medication	Diseases and traits	UKBB	-0.007791	1.30E-06	154702	risk diff			
rs10752269	chr10:12692902	A	G	Pain type experienced in last month: none of the above	Diseases and traits	UKBB	0.006305	1.40E-07	336650	risk diff	0.5368	intron	CAMK1D
				Qualifications: none	Diseases and traits	UKBB	-0.00464	5.12E-07	334070	risk diff			
				Qualifications: college or university degree	Diseases and traits	UKBB	0.005039	9.80E-06	334070	risk diff			
rs10764990	chr10:129152608	A	G								0.6163	intron	DOCK1
rs11164870	chr1:93552187	C	G	Qualifications: college or university degree	Diseases and traits	UKBB	-0.007496	1.60E-10	334070	risk diff	0.3797	intron	MTF2
				Eosinophil count	Diseases and traits	27863252	-0.02332	2.18E-10	173480	IVNT			
				Sum eosinophil basophil counts	Diseases and traits	27863252	-0.02204	2.04E-09	173480	IVNT			
				Eosinophil percentage of white cells	Diseases and traits	27863252	-0.02172	3.22E-09	173480	IVNT			
				Eosinophil percentage of granulocytes	Diseases and traits	27863252	-0.02054	2.50E-08	173480	IVNT			

				Comparative height size at age 10	Diseases and traits	UKBB	0.009217	6.64E-08	332021	-		
				Neutrophil percentage of granulocytes	Diseases and traits	27863252	0.01935	1.51E-07	173480	IVNT		
				Monocyte count	Diseases and traits	27863252	-0.01902	2.31E-07	173480	IVNT		
				Home area population density: Scotland large urban area	Diseases and traits	UKBB	-0.002728	4.16E-07	333997	risk diff		
				Height	Diseases and traits	UKBB	0.008557	1.46E-06	336474	IVNT		
				Monocyte percentage of white cells	Diseases and traits	27863252	-0.01705	3.32E-06	173480	IVNT		
rs11587444	chr1:150722844	A	G	Granulocyte percentage of myeloid white cells	Diseases and traits	27863252	0.03801	2.71E-25	173480	IVNT	0.5915	intron	CTSS
				Monocyte percentage of white cells	Diseases and traits	27863252	-0.03649	1.41E-23	173480	IVNT		
				Monocyte count	Diseases and traits	27863252	-0.0346	3.01E-21	173480	IVNT		
				Neutrophil percentage of white cells	Diseases and traits	27863252	0.0282	1.18E-14	173480	IVNT		
				Forced vital capacity	Diseases and traits	UKBB	-0.01181	5.21E-09	307638	IVNT		
				Forced vital capacity, best measure	Diseases and traits	UKBB	-0.01221	3.78E-08	255492	IVNT		
				Lymphocyte percentage of white cells	Diseases and traits	27863252	-0.01973	6.36E-08	173480	IVNT		
				Lymphocyte count	Diseases and traits	27863252	-0.01856	4.79E-07	173480	IVNT		
				Impedance of arm right	Diseases and traits	UKBB	0.008469	1.31E-06	331279	IVNT		
				Eosinophil percentage of granulocytes	Diseases and traits	27863252	-0.01767	1.41E-06	173480	IVNT		

				Neutrophil percentage of granulocytes	Diseases and traits	27863252	0.01719	2.73E-06	173480	IVNT			
				Impedance of whole body	Diseases and traits	UKBB	0.008671	4.92E-06	331284	IVNT			
rs2117137	chr3:89525505	G	A								0.4026	intron	EPHA3
rs17245213	chr11:1679769	A	G								0.2296	downstream	AP006285.7
rs9302428	chr16:24717600	C	G								0.3718	intron	TNRC6A
rs2273447	chr20:62900120	A	T								0.8022	intron	PCMTD2
rs4817505	chr21:34343828	C	T								0.3807	intron	AP000282.2
rs9624470	chr22:24820268	A	G	Creatinine in urine	Diseases and traits	UKBB	-0.01292	3.35E-08	327525	IVNT	0.5895	intron	ADORA2A
				Sodium in urine	Diseases and traits	UKBB	-0.01169	1.18E-06	326831	IVNT			
rs1481012	chr4:89039082	A	G								0.9105	intron	ABCG2
rs72797284	chr5:152031650	A	G	Getting up in morning	Diseases and traits	UKBB	-0.01332	2.00E-10	336501	-	0.7396	intron	LINC01470
				Morning or evening person	Diseases and traits	UKBB	0.01672	4.68E-10	301143	-			
				Qualifications: college or university degree	Diseases and traits	UKBB	0.006286	8.84E-07	334070	risk diff			
				Comparative body size at age 10	Diseases and traits	UKBB	0.009053	1.24E-06	331693	-			
				Schizophrenia	Diseases and traits	25056061	0.0576	3.05E-06	82315	log OR			
				Loneliness, isolation	Diseases and traits	UKBB	0.004708	7.06E-06	332263	risk diff			
rs7757102	chr6:137222671	A	G	Sum neutrophil eosinophil counts	Diseases and traits	27863252	0.01687	2.71E-06	173480	IVNT	0.4245	intron	PEX7
				Granulocyte count	Diseases and traits	27863252	0.01657	4.19E-06	173480	IVNT			
				Neutrophil count	Diseases and traits	27863252	0.01645	4.64E-06	173480	IVNT			

				Myeloid white cell count	Diseases and traits	27863252	0.01653	4.66E-06	173480	IVNT			
				Sum basophil neutrophil counts	Diseases and traits	27863252	0.01617	7.00E-06	173480	IVNT			
rs57462170	chr3:50239803	A	G	Impedance of leg right	Diseases and traits	UKBB	0.0274	7.70E-15	331301	IVNT	0.1163	upstream	SLC38A3
				Impedance of leg left	Diseases and traits	UKBB	0.02724	1.75E-14	331296	IVNT			
				Impedance of whole body	Diseases and traits	UKBB	0.02085	2.22E-12	331284	IVNT			
				Impedance of arm right	Diseases and traits	UKBB	0.01608	4.40E-09	331279	IVNT			
				Neuroticism score	Diseases and traits	UKBB	0.07933	1.31E-08	274108	-			
				Worrier or anxious feelings	Diseases and traits	UKBB	0.01059	4.39E-08	328717	risk diff			
				High light scatter percentage of red cells	Diseases and traits	27863252	-0.03162	4.62E-08	173480	IVNT			
				Nervous feelings	Diseases and traits	UKBB	0.00887	1.20E-07	328725	risk diff			
				Getting up in morning	Diseases and traits	UKBB	-0.01576	1.40E-07	336501	-			
				Impedance of arm left	Diseases and traits	UKBB	0.01438	1.73E-07	331292	IVNT			
				High light scatter reticulocyte count	Diseases and traits	27863252	-0.03001	2.14E-07	173480	IVNT			
				Immature fraction of reticulocytes	Diseases and traits	27863252	-0.02945	2.77E-07	173480	IVNT			
				Tense or highly strung	Diseases and traits	UKBB	0.007602	3.51E-07	327232	risk diff			
				Comparative body size at age 10	Diseases and traits	UKBB	-0.01303	1.03E-06	331693	-			

				Frequency of depressed mood in last 2 weeks	Diseases and traits	UKBB	0.01117	3.37E-06	323267	-			
				Reticulocyte fraction of red cells	Diseases and traits	27863252	-0.02654	4.63E-06	173480	IVNT			
rs2279844	chr17:40819809										0.4225	downstream	PLEKHH3
rs57631352	chr19:4338173	A	G	Morning or evening person	Diseases and traits	UKBB	-0.01168	8.21E-06	301143	-	0.7137	intron	STAP2
rs1156588	chr2:58515375	G	A								0.1829	intron	LINC01795
rs17685	chr7:75616105	G	A	Coffee consumption cups per day	Diseases and traits	25288136	-0.07	4.00E-11	-	unit increase	0.7107	3_prime_UTR	POR
				Mean platelet volume	Diseases and traits	27863252	0.02567	1.68E-10	173480	IVNT			
				Coffee consumption	Diseases and traits	25288136	-0.08	1.00E-09	-	unit increase			
				Creatinine in urine	Diseases and traits	UKBB	0.01514	3.50E-09	327525	IVNT			
				Eosinophil count	Diseases and traits	27863252	0.02061	2.02E-07	173480	IVNT			
				Eosinophil percentage of white cells	Diseases and traits	27863252	0.0199	5.09E-07	173480	IVNT			
				Sum eosinophil basophil counts	Diseases and traits	27863252	0.01942	1.00E-06	173480	IVNT			
rs2472297	chr15:75027880	C	T	Creatinine in urine	Diseases and traits	UKBB	0.02714	1.42E-25	327525	IVNT	0.7853	intergenic	CYP1A1
				Coffee consumption cups per day	Diseases and traits	25288136	-0.14	2.00E-24	-	unit increase			
				Caffeine metabolism plasma 17 dimethylxanthine paraxanthine to 137 trimethylxanthine caffeine ratio	Diseases and traits	27702941	-9.58	9.00E-22	-	Z-score increase			
				Caffeine metabolism plasma 137 trimethylxanthine caffeine level	Diseases and traits	27702941	-9.34	1.00E-20	-	Z-score decrease			
				Sodium in urine	Diseases and traits	UKBB	0.02029	2.98E-14	326831	IVNT			

				Coffee consumption	Diseases and traits	21357676	-0.31	5.00E-14	-	increase in cups per day			
				Coffee	Diseases and traits	21357676	NA	5.00E-14	-	-			
				Coffee consumption	Diseases and traits	21357676	NA	5.40E-14	6611	-			
				Coffee consumption	Diseases and traits	21876539	NA	2.70E-11	18176	-			
rs56188862	chr1:174189269	C	T	Medication for pain relief, constipation, heartburn: none of the above	Diseases and traits	UKBB	0.007475	2.33E-09	333581	risk diff	0.3837	intron	RABGAP1L
				Treatment with paracetamol	Diseases and traits	UKBB	-0.005636	5.41E-09	337159	risk diff			
				Tense or highly strung	Diseases and traits	UKBB	-0.005534	7.31E-09	327232	risk diff			
				Medication for pain relief, constipation, heartburn: paracetamol	Diseases and traits	UKBB	-0.005831	1.75E-08	333581	risk diff			
				Neuroticism score	Diseases and traits	UKBB	-0.04275	1.77E-06	274108	-			
				Number of treatments or medications taken	Diseases and traits	UKBB	-0.009425	2.56E-06	337159	-			
				Pain type experienced in last month: none of the above	Diseases and traits	UKBB	0.00568	3.96E-06	336650	risk diff			
				Pain type experienced in last month: knee pain	Diseases and traits	UKBB	-0.004716	4.34E-06	336650	risk diff			
rs9648476	chr7:39293033	A	G								0.6143	intron	POU6F2
rs4410790	chr7:17284577	C	T	Habitual caffeine consumption caffeinated coffee intake	Diseases and traits	21490707	NA	1.40E-29	47431	-	0.6153	intergenic	AC003075.4
				Caffeine consumption	Diseases and traits	21490707	-0.15	2.00E-19	-	mg/day decrease			
				Caffeine	Diseases and traits	21490707	NA	2.00E-19	-	-			
				Habitual caffeine consumption	Diseases and traits	21490707	NA	2.36E-19	47431	-			

				Creatinine in urine	Diseases and traits	UKBB	-0.02103	1.32E-18	327525	IVNT			
				Coffee consumption cups per day	Diseases and traits	25288136	-0.1	3.00E-17	-	unit decrease			
				Habitual caffeine consumption female	Diseases and traits	21490707	NA	2.40E-16	47431	-			
				Caffeine metabolism plasma 17 dimethylxanthine paraxanthine to 137 trimethylxanthine caffeine ratio	Diseases and traits	27702941	-8.07	7.00E-16	-	Z-score decrease			
				Habitual caffeine consumption never smokers	Diseases and traits	21490707	NA	1.80E-14	47431	-			
				Caffeine metabolism plasma 137 trimethylxanthine caffeine level	Diseases and traits	27702941	-7.36	2.00E-13	-	Z-score increas			
rs12591786	chr15:60902512	C	T	Hip circumference	Diseases and traits	UKBB	0.0164	9.58E-07	336601	IVNT	0.827	intron	RORA
rs4808193	chr19:19410622	C	T	Plateletcrit	Diseases and traits	27863252	0.03479	3.34E-19	173480	IVNT	0.3191	intron	SUGP1
				Impedance of leg left	Diseases and traits	UKBB	0.01719	2.51E-13	331296	IVNT			
				Impedance of leg right	Diseases and traits	UKBB	0.01697	3.27E-13	331301	IVNT			
				Red cell distribution width	Diseases and traits	27863252	-0.02663	1.37E-12	173480	IVNT			
				Platelet count	Diseases and traits	27863252	0.02594	2.05E-11	173480	IVNT			
				Self-reported high cholesterol	Diseases and traits	UKBB	-0.004798	1.33E-08	337159	risk diff			
				Comparative body size at age 10	Diseases and traits	UKBB	-0.01001	1.37E-08	331693	-			
				Basophil count	Diseases and traits	27863252	0.02101	1.49E-08	173480	IVNT			

				Impedance of whole body	Diseases and traits	UKBB	0.01071	5.03E-08	331284				
rs13282783	chr8:22088975	C	T								0.66	intron	PHYHIP
rs2351187	chr10:86850616	G	A								0.6819	intergenic	LINC01519
rs1453548	chr11:59192089	A	T	Ever smoked	Diseases and traits	UKBB	0.007159	1.43E-08	336067	risk diff	0.6909	upstream	OR5A2
				Past tobacco smoking	Diseases and traits	UKBB	-0.01743	2.15E-07	310749	-			
				Smoking status: previous	Diseases and traits	UKBB	0.005931	1.57E-06	336024	risk diff			
rs34619	chr5:60465365	G	A	Qualifications: college or university degree	Diseases and traits	UKBB	-0.009179	1.28E-15	334070	risk diff	0.5875	intron	SMIM15-AS1
				Qualifications: none	Diseases and traits	UKBB	0.007118	2.05E-14	334070	risk diff			
				Years of educational attainment	Diseases and traits	27225129	-0.016	1.62E-10	328917	years			
				Frequency of stair climbing in last 4 weeks	Diseases and traits	UKBB	-0.01845	7.32E-09	334169	-			
				Time spent watching television	Diseases and traits	UKBB	0.009976	1.21E-07	319740	-			
				Qualifications: A levels or as levels or equivalent	Diseases and traits	UKBB	-0.005816	1.26E-07	334070	risk diff			
rs713598	chr7:141673345	G	C	Propylthiouracil PROP solution taste intensity	Diseases and traits	20675712	NA	1.60E-104	1457	-	0.4225	missense	TAS2R38
				Bitter taste response	Diseases and traits	20675712	NA	2.00E-104	-	-			
				Hemoglobin a glycosylated	Diseases and traits	20675712	NA	2.00E-104	-	-			
				PROP taste detection threshold	Diseases and traits	22132133	NA	1.21E-52	225	-			
				Propylthiouracil PROP paper taste intensity	Diseases and traits	20675712	NA	2.20E-42	1457	-			

				Treatment with chlorphenamine	Diseases and traits	UKBB	-0.000289	1.90E-06	337159	risk diff			
				Bipolar disorder	Diseases and traits	27329760	-0.1121	5.42E-06	40255	log			
rs2478875	chr6:51283110	A	G	Sodium in urine	Diseases and traits	UKBB	0.01924	3.87E-11	326831	IVNT	0.7913	intergenic	RP11-228O6.2
				Morning or evening person	Diseases and traits	UKBB	-0.01521	2.34E-07	301143	-			
rs56348300	chr9:7054124	C	G	Comparative height size at age 10	Diseases and traits	UKBB	-0.01015	2.10E-06	332021	-	0.831	intron	KDM4C
rs2783129	chr13:80168720	C	G								0.496	intergenic	LINC01068
rs2645929	chr13:56444529	G	A								0.8728	intergenic	HNF4GP1
rs17576658	chr13:100272019	A	G								0.2604	intron	CLYBL
rs132904	chr22:41798896	C	G	Neutrophil percentage of granulocytes	Diseases and traits	27863252	0.02863	3.23E-11	173480	IVNT	0.7744	downstream	TEF
				Eosinophil percentage of granulocytes	Diseases and traits	27863252	-0.02762	1.53E-10	173480	IVNT			
				Allergic disease	Diseases and traits	29083406	-0.0436	3.10E-10	360838	log OR			
				Impedance of arm left	Diseases and traits	UKBB	-0.0129	4.65E-10	331292	IVNT			
				Eosinophil percentage of white cells	Diseases and traits	27863252	-0.02546	3.08E-09	173480	IVNT			
				Sum eosinophil basophil counts	Diseases and traits	27863252	-0.02448	1.29E-08	173480	IVNT			
				Impedance of arm right	Diseases and traits	UKBB	-0.01172	1.29E-08	331279	IVNT			
				No blood clot, bronchitis, emphysema, asthma, rhinitis, eczema or allergy diagnosed by doctor	Diseases and traits	UKBB	0.007607	3.01E-08	336782	risk diff			
				Eosinophil count	Diseases and traits	27863252	-0.02353	4.46E-08	173480	IVNT			

rs977474	chr12:11284772	T	C								0.8678	intron	PRR4
rs149805207	chr6:137095269	A	G								0.9871	intron	MAP3K5
rs141071726	chr7:17558580	A	G								0.0179	intron	AC017060.1
rs9937354	chr16:53799847	A	G								0.4443	intron	FTO
rs6829	chr13:111531264	T	C	Home area population density: Scotland large urban area	Diseases and traits	UKBB	0.002438	5.31E-06	333997	risk diff	0.6133	3_prime_UTR	ANKRD10
				Longest period of depression	Diseases and traits	UKBB	-0.02993	5.71E-06	46884	IVNT			

Table S3. Instrumental variants (IVs) details in MR analyses. In the forward MR analysis, tea intake was considered as the exposure and gout risk as the outcome. Conversely, in reverse MR analysis, gout risk was regarded as the exposure and tea intake as the outcome.

								Exposure			Outcome					
Exposure	Outcome	IVs	effect_allele.exposure	other_allele.exposure	effect_allele.outcome	other_allele.outcome	Beta	SE	p Value	Beta	SE	p Value	chr	pos	F	R2
Forward MR																
Tea intake	Gout, unspecified (ukb-b-12765)	rs10741694	C	T	C	T	0.0150037	0.002194	7.90E-12	-0.0001083	0.000102	0.29	11	16286183	46.78446	0.000105
		rs10752269	A	G	A	G	-0.0128727	0.00212	1.30E-09	-1.751E-05	9.853E-05	0.86	10	12692902	36.87822	8.24E-05
		rs10764990	A	G	A	G	-0.0121906	0.002169	1.90E-08	1.619E-05	0.0001008	0.87	10	1.29E+08	31.58923	7.06E-05
		rs11164870	G	C	G	C	-0.0119604	0.002182	4.20E-08	0.0001021	0.0001015	0.31	1	93552187	30.03686	6.71E-05
		rs11587444	G	A	G	A	0.0140328	0.002171	1.00E-10	-0.0001405	0.000101	0.16	1	1.51E+08	41.78853	9.34E-05
		rs2117137	G	A	G	A	0.0129948	0.002156	1.70E-09	-0.000114	0.0001002	0.26	3	89525505	36.33813	8.12E-05
		rs2279844	A	G	A	G	-0.0119879	0.002183	4.00E-08	3.043E-05	0.0001014	0.760001	17	40819809	30.15138	6.74E-05
		rs34619	A	G	A	G	0.0117117	0.002138	4.30E-08	-0.0001069	9.936E-05	0.28	5	60465365	30.02117	6.71E-05
		rs4410790	C	T	C	T	0.0405506	0.002195	3.40E-76	-0.0001382	0.000102	0.18	7	17284577	341.2698	0.000762
		rs4817505	C	T	C	T	0.015068	0.002175	4.20E-12	-3.824E-05	0.000101	0.709999	21	34343828	48.01229	0.000107
		rs56188862	C	T	C	T	-0.0157568	0.002175	4.30E-13	8.927E-05	0.0001011	0.38	1	1.74E+08	52.49734	0.000117
		rs6829	T	C	T	C	-0.0119163	0.002165	3.70E-08	3.953E-05	0.0001007	0.69	13	1.12E+08	30.28186	6.77E-05
		rs713598	G	C	G	C	0.0133969	0.002157	5.20E-10	0.000152	0.0001003	0.13	7	1.42E+08	38.58988	8.62E-05
		rs7757102	G	A	G	A	-0.0118039	0.002133	3.10E-08	6.959E-05	9.918E-05	0.48	6	1.37E+08	30.62395	6.84E-05
		rs9302428	G	C	G	C	0.0122457	0.002201	2.60E-08	5.959E-05	0.0001023	0.56	16	24717600	30.94855	6.92E-05
		rs9624470	A	G	A	G	0.0252071	0.002155	1.30E-31	-9.477E-05	0.0001001	0.34	22	24820268	136.8396	0.000306
		rs9648476	A	G	A	G	0.0125013	0.002185	1.10E-08	-6.538E-06	0.0001016	0.95	7	39293033	32.72205	7.31E-05
		rs9937354	A	G	A	G	-0.0140923	0.002143	4.90E-11	5.078E-05	9.959E-05	0.61	16	53799847	43.23125	9.66E-05
	Gout(finn-b-M13_GOUT)	rs11164870	G	C	G	C	-0.0119604	0.002182	4.20E-08	0.0243	0.0261	0.3521	1	93552187	30.03686	6.71E-05
		rs1156588	G	A	G	A	-0.015454	0.002603	2.90E-09	-0.0368	0.0302	0.223	2	58515375	35.24115	7.87E-05
		rs11587444	G	A	G	A	0.0140328	0.002171	1.00E-10	-0.0111	0.0257	0.6651	1	1.51E+08	41.78853	9.34E-05
		rs12591786	T	C	T	C	-0.0184399	0.002942	3.70E-10	0.0093	0.0423	0.8253	15	60902512	39.27397	8.78E-05
		rs13282783	T	C	T	C	-0.0135837	0.002354	7.90E-09	-0.0186	0.0258	0.4701	8	22088975	33.28934	7.44E-05
		rs132904	C	G	C	G	0.0166007	0.002553	7.80E-11	-0.0267	0.0295	0.366	22	41798896	42.29582	9.45E-05

	rs141071726	A	G	A	G	0.0407321	0.006812	2.20E-09	-0.0472	0.108	0.662	7	17558580	35.75355	7.99E-05
	rs1453548	A	T	A	T	-0.0133414	0.00225	3.00E-09	0.0047	0.0267	0.859	11	59192089	35.16754	7.86E-05
	rs149805207	G	A	G	A	-0.0719337	0.012582	1.10E-08	0.108	0.0915	0.238	6	1.37E+08	32.68472	0.000073
	rs17245213	A	G	A	G	-0.0146481	0.002609	2.00E-08	0.025	0.0331	0.4499	11	1679769	31.52084	7.04E-05
	rs17576658	A	G	A	G	-0.0134812	0.002457	4.10E-08	-0.0079	0.0304	0.7959	13	1E+08	30.1166	6.73E-05
	rs17685	A	G	A	G	0.0230655	0.002362	1.60E-22	-0.0073	0.026	0.778599	7	75616105	95.36398	0.000213
	rs2273447	T	A	T	A	0.0174715	0.002634	3.30E-11	-0.0282	0.0369	0.4439	20	62900120	43.99057	9.83E-05
	rs2279844	A	G	A	G	-0.0119879	0.002183	4.00E-08	-0.0371	0.0261	0.1555	17	40819809	30.15138	6.74E-05
	rs2351187	A	G	A	G	0.0129023	0.002282	1.60E-08	0.0117	0.0272	0.666201	10	86850616	31.95868	7.14E-05
	rs2472297	T	C	T	C	0.0533453	0.002401	2.30E-109	-0.0785	0.0294	0.007583	15	75027880	493.6456	0.001102
	rs2478875	G	A	G	A	0.0218943	0.002611	5.10E-17	-0.0269	0.0333	0.4205	6	51283110	70.29945	0.000157
	rs2645929	G	A	G	A	-0.0149842	0.002717	3.50E-08	-0.0423	0.035	0.2275	13	56444529	30.42396	0.000068
	rs34619	A	G	A	G	0.0117117	0.002138	4.30E-08	0.0503	0.0256	0.0493503	5	60465365	30.02117	6.71E-05
	rs4808193	C	T	C	T	0.0151149	0.002247	1.70E-11	0.0057	0.0293	0.8469	19	19410622	45.24047	0.000101
	rs56188862	C	T	C	T	-0.0157568	0.002175	4.30E-13	0.0006	0.0261	0.9818	1	1.74E+08	52.49734	0.000117
	rs56348300	G	C	G	C	0.0158824	0.002732	6.10E-09	-0.0084	0.0377	0.8236	9	7054124	33.79866	7.55E-05
	rs57462170	A	G	A	G	0.0191505	0.003406	1.90E-08	0.0828	0.0364	0.0229398	3	50239803	31.62025	7.07E-05
	rs57631352	G	A	G	A	-0.0131035	0.002321	1.70E-08	-0.017	0.0268	0.5256	19	4338173	31.86843	7.12E-05
	rs6829	T	C	T	C	-0.0119163	0.002165	3.70E-08	0.0225	0.0257	0.3806	13	1.12E+08	30.28186	6.77E-05
	rs72797284	G	A	G	A	-0.0171147	0.002384	7.00E-13	0.079	0.031	0.01078	5	1.52E+08	51.55815	0.000115
	rs7757102	G	A	G	A	-0.0118039	0.002133	3.1E-08	-0.0001	0.0255	0.9972	6	1.37E+08	30.62395	6.84E-05
	rs9302428	G	C	G	C	0.0122457	0.002201	2.6E-08	-0.0287	0.0262	0.2734	16	24717600	30.94855	6.92E-05
	rs9624470	A	G	A	G	0.0252071	0.002155	1.3E-31	-0.042	0.0254	0.0978093	22	24820268	136.8396	0.000306
	rs9648476	A	G	A	G	0.0125013	0.002185	1.1E-08	-0.0069	0.0263	0.7942	7	39293033	32.72205	7.31E-05
	rs977474	T	C	T	C	0.0217813	0.002856	2.4E-14	-0.0237	0.0451	0.5989	12	11284772	58.18029	0.00013
	rs9937354	A	G	A	G	-0.0140923	0.002143	4.9E-11	0.0406	0.0255	0.1113	16	53799847	43.23125	9.66E-05
	rs10741694	C	T	C	T	0.0150037	0.002194	7.90E-12	-0.0301	0.0399	0.4509	11	16286183	43.23125	9.66E-05
	rs10752269	A	G	A	G	-0.0128727	0.00212	1.30E-09	-0.0387	0.037	0.2955	10	12692902	0.000105	46.78446
	rs10764990	A	G	A	G	-0.0121906	0.002169	1.90E-08	-0.0026	0.0355	0.9404	10	1.29E+08	8.24E-05	36.87822
Gout, strict definition(finn-b-GOUT_STRICT)	rs11164870	G	C	G	C	-0.0119604	0.002182	4.20E-08	0.0306	0.0365	0.4011	1	93552187	7.06E-05	31.58923
	rs1156588	G	A	G	A	-0.015454	0.002603	2.90E-09	-0.0762	0.0422	0.07087	2	58515375	6.71E-05	30.03686

rs11587444	G	A	G	A	0.0140328	0.002171	1.00E-10	0.0081	0.0359	0.8219	1	1.51E+08	7.87E-05	35.24115
rs12591786	T	C	T	C	-0.0184399	0.002942	3.70E-10	0.0154	0.059	0.7949	15	60902512	9.34E-05	41.78853
rs13282783	T	C	T	C	-0.0135837	0.002354	7.90E-09	-0.0581	0.0362	0.1085	8	22088975	8.78E-05	39.27397
rs132904	C	G	C	G	0.0166007	0.002553	7.80E-11	0.015	0.0413	0.716	22	41798896	7.44E-05	33.28934
rs141071726	A	G	A	G	0.0407321	0.006812	2.20E-09	-0.1213	0.1502	0.4193	7	17558580	9.45E-05	42.29582
rs1453548	A	T	A	T	-0.0133414	0.00225	3.00E-09	-0.0464	0.0374	0.2149	11	59192089	7.99E-05	35.75355
rs149805207	G	A	G	A	-0.0719337	0.012582	1.10E-08	0.1891	0.1286	0.1414	6	1.37E+08	7.86E-05	35.16754
rs17245213	A	G	A	G	-0.0146481	0.002609	2.00E-08	0.0179	0.0465	0.6996	11	1679769	0.000073	32.68472
rs17576658	A	G	A	G	-0.0134812	0.002457	4.10E-08	-0.021	0.0424	0.6209	13	1E+08	7.04E-05	31.52084
rs17685	A	G	A	G	0.0230655	0.002362	1.60E-22	-0.0088	0.0363	0.8087	7	75616105	6.73E-05	30.1166
rs2273447	T	A	T	A	0.0174715	0.002634	3.30E-11	-0.0224	0.0517	0.6643	20	62900120	0.000213	95.36398
rs2279844	A	G	A	G	-0.0119879	0.002183	4.00E-08	-0.0845	0.0366	0.02088	17	40819809	9.83E-05	43.99057
rs2351187	A	G	A	G	0.0129023	0.002282	1.60E-08	0.0178	0.0381	0.639401	10	86850616	6.74E-05	30.15138
rs2472297	T	C	T	C	0.0533453	0.002401	#######	-0.0963	0.0409	0.01864	15	75027880	7.14E-05	31.95868
rs2478875	G	A	G	A	0.0218943	0.002611	5.10E-17	-0.0247	0.0467	0.5966	6	51283110	0.001102	493.6456
rs2645929	G	A	G	A	-0.0149842	0.002717	3.50E-08	0.0233	0.049	0.6351	13	56444529	0.000157	70.29945
rs34619	A	G	A	G	0.0117117	0.002138	4.30E-08	0.0321	0.0358	0.3709	5	60465365	6.76E-05	30.25428
rs4410790	C	T	C	T	0.0405506	0.002195	3.40E-76	-0.0516	0.0375	0.1684	7	17284577	6.71E-05	30.02117
rs4808193	C	T	C	T	0.0151149	0.002247	1.70E-11	0.0287	0.041	0.483499	19	19410622	0.000762	341.2698
rs4817505	C	T	C	T	0.015068	0.002175	4.20E-12	-0.0051	0.0358	0.8872	21	34343828	0.000101	45.24047
rs56188862	C	T	C	T	-0.0157568	0.002175	4.30E-13	-0.0103	0.0367	0.7782	1	1.74E+08	0.000107	48.01229
rs56348300	G	C	G	C	0.0158824	0.002732	6.10E-09	-0.0339	0.0527	0.5198	9	7054124	0.000117	52.49734
rs57631352	G	A	G	A	-0.0131035	0.002321	1.70E-08	-0.0126	0.0374	0.7358	19	4338173	7.55E-05	33.79866
rs6829	T	C	T	C	-0.0119163	0.002165	3.70E-08	0.1057	0.036	0.003286	13	1.12E+08	7.12E-05	31.86843
rs713598	G	C	G	C	0.0133969	0.002157	5.20E-10	0.0212	0.037	0.5673	7	1.42E+08	6.77E-05	30.28186
rs72797284	G	A	G	A	-0.0171147	0.002384	7.00E-13	0.0964	0.0433	0.02613	5	1.52E+08	8.62E-05	38.58988
rs7757102	G	A	G	A	-0.0118039	0.002133	3.10E-08	0.0482	0.0356	0.1761	6	1.37E+08	0.000115	51.55815
rs9302428	G	C	G	C	0.0122457	0.002201	2.60E-08	0.0006	0.0367	0.9877	16	24717600	6.84E-05	30.62395
rs9624470	A	G	A	G	0.0252071	0.002155	1.30E-31	-0.0377	0.0354	0.2874	22	24820268	0.000306	136.8396
rs9648476	A	G	A	G	0.0125013	0.002185	1.10E-08	-0.048	0.0367	0.1907	7	39293033	7.31E-05	32.72205
rs977474	T	C	T	C	0.0217813	0.002856	2.40E-14	0.0279	0.0635	0.6605	12	11284772	0.00013	58.18029
rs9937354	A	G	A	G	-0.0140923	0.002143	4.90E-11	0.0134	0.0356	0.705601	16	53799847	9.66E-05	43.23125

		rs2725245	A	G	A	G	-0.00075	0.000101	1.1E-13	-0.00534	0.002125	0.012	4	89068738	55.15145	0.000119
		rs10805346	C	T	C	T	-0.00061	9.96E-05	8.4E-10	0.001332	0.002141	0.53	4	9920347	37.65452	8.13E-05
		rs4665972	C	T	C	T	0.000875	9.88E-05	8.5E-19	0.004224	0.002176	0.052	2	27598097	78.3883	0.000169
**Reverse MR**																
**Gout, unspecified**	**Tea intake**	rs34004016	C	T	C	T	0.001795	0.002484	0.47	0.001795	0.002484	0.47	4	9697965	33.53993	0.000003
**(ukb-b-12765)**																
		rs3775948	C	G	C	G	-0.00407	0.00246	0.098001	-0.00407	0.00246	0.098001	4	9995182	189.4003	0.999998
		rs4645896	A	G	A	G	-0.0005	0.004305	0.91	-0.0005	0.004305	0.91	19	49461834	143.3649	0.269583
**Gout(finn-b-**	**Tea intake**	rs149136965	C	G	C	G	0.01558	0.009287	0.092999	0.01558	0.009287	0.092999	6	30958409	30.98934	0.486652
**M13_GOUT)**																
		rs1064257	G	C	G	C	0.002065	0.004762	0.66	0.002065	0.004762	0.66	19	49993535	38.89569	0.999986
		rs112395288	T	C	T	C	0.017968	0.014424	0.21	0.017968	0.014424	0.21	19	50505241	126.5071	0.999922
		rs2581792	C	T	C	T	0.2141	0.0393	4.93E-08	0.001795	0.002484	0.47	4	9697965	29.67893	0.384927
		rs34004016	C	T	C	T	-0.2193	0.0389	1.66E-08	-0.00407	0.00246	0.098001	4	9995182	31.78177	0.441327
		rs3775948	C	G	C	G	0.4363	0.0426	1.33E-24	0.001371	0.002433	0.57	6	25824809	104.8941	0.955441
**Gout, strict**	**Tea intake**	rs9467618	T	C	T	C	0.2343	0.0427	4.21E-08	0.001958	0.002339	0.4	3	53035044	30.10848	0.198602
**definition(finn-b-**																
**GOUT_STRICT)**																
		rs1064257	G	C	G	C	1.1029	0.1317	5.47E-17	0.002065	0.004762	0.66	19	49993535	70.1295	0.999933
		rs112395288	T	C	T	C	0.8612	0.1414	1.12E-09	0.017968	0.014424	0.21	19	50505241	37.09447	0.999915

IVs, instrumental variants ; MR, Mendelian randomization; SE, standard error.

Table S4. The Heterogeneity and Pleiotropy test of Reverse MR analysis. In the reverse MR analysis, gout risk was regarded as the exposure and tea intake as the outcome.

		Heterogeneity test		Pleiotropy test	
**Exprosure**	**Outcome**	**IVW p**	**MR - Egger p**	**MR - Egger *p***	**PRESSO *p***
Gout, unspecified (ukb-b-12765)	Tea intake	0.6377	0.775	0.5319	0.5230
Gout(finn-b-M13_GOUT)		0.1697	0.2012	0.3349	0.3220
Gout, strict definition(finn-b-GOUT_STRICT)		0.3071	0.2024	0.8863	0.3840

IVW, inverse variance weighted; MR, Mendelian randomization;

Table S5. Reverse MR analysis of exposures (Gout risk) with outcome (Tea intake).

**Exposure**	**Outcome**	**Method**	**nsnp**	**b**	**SE**	**p value**	**OR**	**Lower 95% (**	**Upper 95% (**
Gout, unspecified (ukb-b-12765)	Tea intake(ukb-b-6066)	MR Egger	3	-15.32075953	11.4339622	0.408157453	2.21962E-07	4.10671E-17	1199.675503
		Weighted median	3	-5.760507412	1.947898945	0.003103533	0.003149513	6.92083E-05	0.143327153
		Inverse variance weighted	3	-5.08783497	1.6437671	0.001966612	0.006171367	0.000246138	0.154733429
		Simple mode	3	-5.86575382	2.267786208	0.122584778	0.002834885	3.3278E-05	0.241498298
		Weighted mode	3	-5.921042813	2.356206203	0.128524615	0.002682401	2.64777E-05	0.271748396
Gout(finn-b-M13_GOUT)	Tea intake(ukb-b-6066)	MR Egger	6	0.005934906	0.007452733	0.470424433	1.005952553	0.991365046	1.020754708
		Weighted median	6	0.000260172	0.004023331	0.948440006	1.000260206	0.992403445	1.008179168
		Inverse variance weighted	6	-0.000829774	0.004253766	0.845339537	0.99917057	0.990874735	1.007535859
		Simple mode	6	-0.005264441	0.006953744	0.483139416	0.994749392	0.98128359	1.008399981
		Weighted mode	6	-0.001648615	0.004265295	0.715014558	0.998352743	0.990041325	1.006733935
Gout, strict definition(finn-b-GOUT_STRICT)	Tea intake(ukb-b-6066)	MR Egger	6	0.000367795	0.006472067	0.957407601	1.000367863	0.987758093	1.013138609
		Weighted median	6	-0.001012177	0.003703342	0.784611581	0.998988335	0.99176338	1.006265924
		Inverse variance weighted	6	-0.000449925	0.003240564	0.889575213	0.999550176	0.993221647	1.005919028
		Simple mode	6	0.003557738	0.006381299	0.60120554	1.003564075	0.99109032	1.016194822
		Weighted mode	6	0.000416561	0.004214756	0.925110253	1.000416647	0.992186325	1.008715241

MR, Mendelian randomization; SE, standard error; OR, odds ratio; CI, confidence intervals.
